# Supplementary material for: Genetic Diversity and Population Structure of Jubaea chilensis, an Endemic and Monotype Gender from Chile, Based on SNP Markers
Source: Plants (Basel). 2022 Jul 28;11(15):1959. doi: 10.3390/plants11151959 (PMC9370131; doi:10.3390/plants11151959)
Supplement: Supplementary file 1 [file plants-11-01959-s001.zip › plants-1798411-supplementary.pdf]

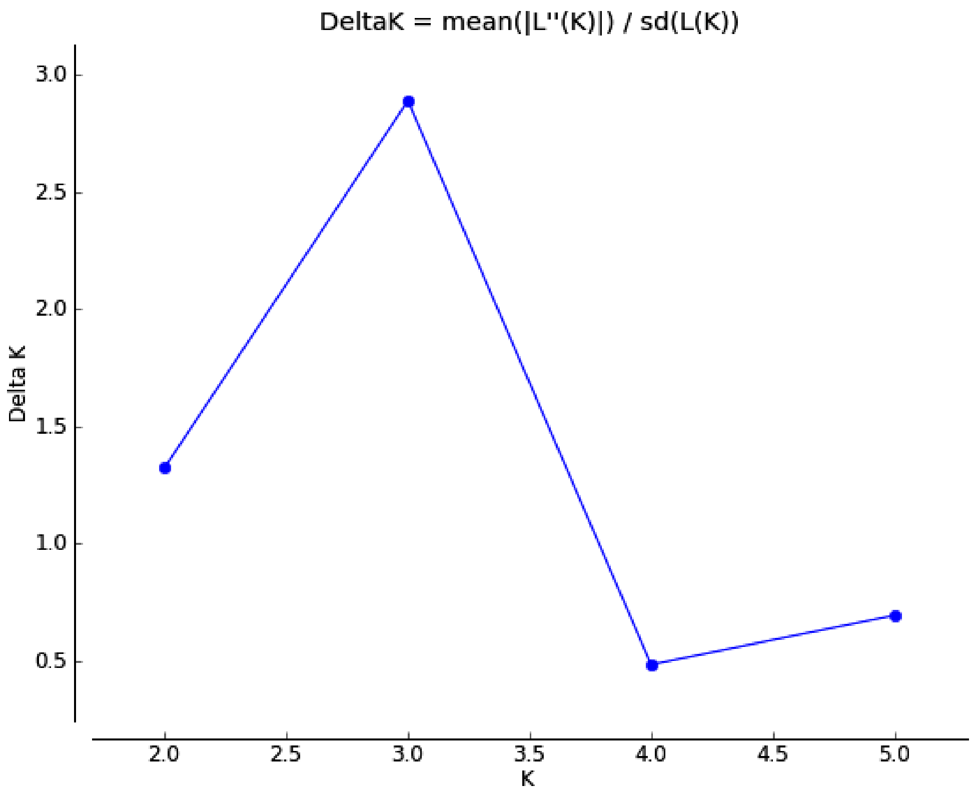

Figure S1. ΔK values for different numbers of populations assumed (K) in the STRUCTURE HARVESTER analysis v 0.6.94 for the six population groups of *Jubaea chilensis*, central Chile.

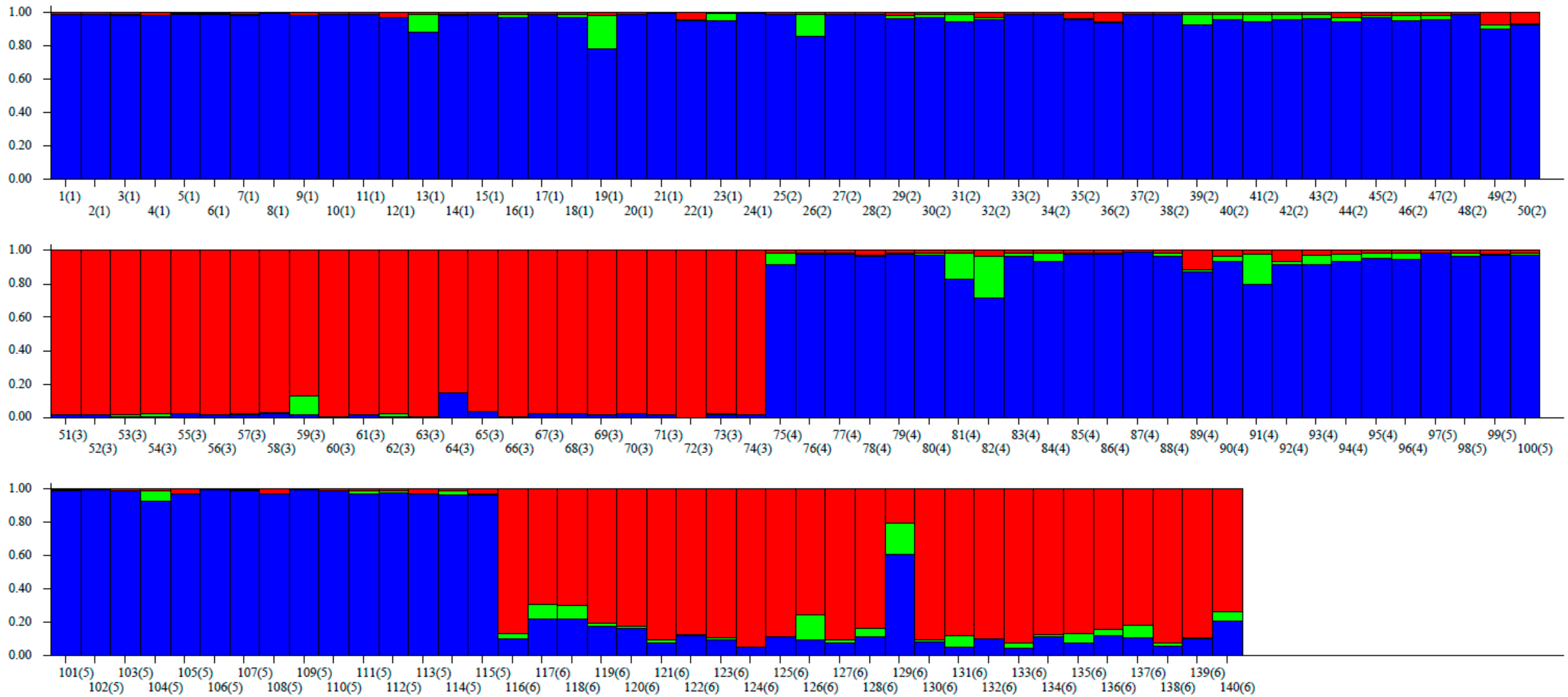

Figure S2. Populational structure inferred for the six sampled groups of *Jubaea chilensis*, central Chile, based on the analysis of 1,038 SNPs using the STRUCTURE v 2.3.4, assuming the Admixture model. Each individual is represented by a vertical bar, often partitioned into colored segments with the length of each segment representing the proportion of the individual's genome from K = 3 ancestral populations. Cocalan:1-24, Culimo:25-50, Viña del Mar/Valparaíso:51-74, Candelaria:75-96, Petorca:97-115, and Ocoa:116-140.

Table S1. Frequency of SNPs by minor allele frequencies (MAF) within the six population groups of *Jubaea chilensis*. (CUL) Culimo, (PET) Petorca, (OCO) Ocoa, (VAV) Viña del Mar/Valparaíso, (COC) Cocalán, (CAN) Candelaria.

	CUL	PET	OCO	VAV	COC	CAN
**MAF ≤ 0.1**	0.994	0.973	0.976	0.969	0.988	0.989
**0.1 < MAF ≤ 0.2**	0.006	0.017	0.022	0.022	0.01	0.01
**0.2 < MAF ≤ 0.3**	0	0.007	0.002	0.007	0.001	0
**0.3 < MAF ≤ 0.4**	0	0.003	0	0.002	0.001	0.001
**0.4 < MAF ≤ 0.5**	0	0	0	0	0	0

Table S2. Log likelihood and Bayesian deviance for BA3-SNPs runs.

Runs	Log likelihood	Deviance
**1**	-17655.7066	35311.41
**2**	-17225.1847	34450.37
**3**	-16909.3281	33818.66
**4**	-17435.152	34870.3
**5**	-17336.3691	34672.74
**6**	-17735.9297	35471.86
**7**	-17306.2556	34612.51
**8**	-17099.8056	34199.61
**9**	-17324.9496	34649.9
**10**	-17342.0692	34684.14

Table S3. Evanno method results (Evanno et al., 2005) obtained using the STRUCTURE HARVESTER v 0.6.94 for the six population groups of *Jubaea chilensis*, central Chile. The highest value of delta K (in bold) indicates the number of established groups.

K	Reps	Mean LnP (K)	Stdev LnP (K)	Ln' (K)	|Ln'' (K)|	Delta K
1	25	-17626.820	1.210	-	-	-
2	25	-16739.548	277.545	887.272	367.980	1.326
**3**	**25**	**-16220.256**	**515.248**	**519.292**	**1488.932**	**2.890**
4	25	-17189.896	2541.272	-969.640	1232.916	0.485
5	25	-16926.620	3753.075	263.276	2608.900	0.695
6	25	-14054.444	874.565	2872.176	-	-
